# Combining the Tait equation with the phonon theory allows predicting the density of liquids up to the Gigapascal range

**DOI:** 10.1038/s41598-023-30917-0

**Published:** 2023-03-07

**Authors:** Eugene B. Postnikov, Roman N. Belenkov, Mirosław Chora̧żewski

**Affiliations:** 1 Department of Theoretical Physics, Kursk State University, Radishcheva st., 33, Kursk, 305000 Russia; 2 Institute of Chemistry, University of Silesia in Katowice, Szkolna 9, 40-006 Katowice, Poland

**Keywords:** Thermodynamics, Thermodynamics

## Abstract

Predicting the density of liquids at ultrahigh pressures in the case when only the data measured at ambient pressure are available is a long-standing challenge for thermodynamic research. In this work, we archived this goal for molecular liquids by applying the half-sum of the Tait equation and the Murnagnan equation in the form coordinated with Tait’s at low pressure for predicting the density of molecular liquids up to the pressures more than 1 GPa with uncertainty comparable with the experimental one. It is shown that the control parameter, which is needed in addition to the initial density and the isothermal compressibility can be found using the speed of sound and the density at ambient pressure and has a clear physical interpretation in terms of the characteristic frequency of intermolecular oscillation mimicking the limiting frequency of Debye’s theory of heat conductivity of solids. This fact is discussed as arguing in favour of the modern phonon theory of liquid thermodynamics and expands it range of applicability to the volumetric properties of liquids at temperatures far below the critical one. The validity of the model is illustrated with the case study of classic Bridgman’s dataset as well as with some examples of ultrahigh-pressure data obtained by the diamond anvil cell and shock wave compression methods.

## Introduction

Knowledge of thermodynamic properties at high-pressure conditions is of permanent research interest not only from the point of view of studying fundamental aspects of molecular properties and intermolecular interactions but also due to prime importance for all chemistry and chemical engineering areas. Therefore, it is not surprising that the experimental determination and modelling of their behaviour have been paramount. For modern physics and chemistry, high pressure, correlated with temperature, is a vital research parameter to understand the thermodynamic, physico-chemical, mechanical, and structural properties of substances under extreme conditions^1^. On the other hand, high pressures applied in materials engineering make it possible to obtain new materials often endowed with distinctive properties^2–4^.

In science and technology, it has become accepted to establish certain numerical ranges of pressure values. The limits of these ranges are conventional and result mainly from established constructional, technological, and testing criteria. The division of pressure into low, medium, high, and ultra-high pressure ranges depends on the state of the substance being investigated. This division varies between fields such as physics, chemistry, biology, and geochemistry and even within one research field. In research on the thermophysical properties of gas, these ranges will be completely different from liquid or solid phase studies. In other words, a pressure considered high in gas phase studies will be called low in liquid or solid phase studies. This is because, in condensed phases like liquids and solids, the pressure depends both on the elastic repulsion forces of the atoms and on changes in the thermal vibrational energy of the atoms that depend on volume changes. In gases, on the other hand, pressure is related to the transfer of momentum by molecules in thermal motion. Nevertheless, from the point of view of molecular physics and physical chemistry of liquid state^5^, the natural limit between high and extremely high pressures can be defined by the value of 1 GPa as close to the maximum possible to a classical thermodynamic system existing in a liquid state before freezing and achievable technically with a piston-cylinder system.

The central development of the technique of generating and measuring high pressures with such technique owes much to the American physicist Percy Williams Bridgman. Given his extraordinary contribution to the development of high-pressure technology, the period of pressure research is often historically divided into the pre-Bridgman, Bridgman years, and post-Bridgman years. He invented the unique system of self-sealing seals based on an uncompensated surface, applying so-called piston-cylinder pressure multipliers with variable diameters to gas–liquid and liquid–liquid compression, and finally, the design of anvil apparatuses resulting from the development of solutions for pressures above 10 GPa^6^. Despite the further development of other tools for creating extreme high-pressures such as diamond anvil cells, shock wave compression, etc., Bridgman’s data remain a gold standard in the field of high-pressure thermodynamics research^7^ since they are obtained for macroscopic samples compressed via the true thermodynamic (reversible) route and widely used as the reference data to testing developed equations of state.

The most common method of representing the thermodynamic properties of liquids is to describe these properties using an appropriate equation of state (EoS). The main roles of EoS are predicting accurate fundamental thermodynamics properties used in the rational design of efficient chemical processes and providing a conceptual molecular-level understanding of thermodynamics properties. However, in contrast to the liquids at thermodynamic states close to the saturation curve, the equations of state reproducing density at high pressures mostly have a phenomenological character with a number of coefficients fitted to reproduce actual high-pressure experimental data^8^ due to complexity of required molecular information, such as intermolecular potential parameters and structural ordering, which prevents the usage of statistical thermodynamics approach. Even sophisticated semi-statistical approaches such as the statistical association fluid theory (SAFT) and its modifications require adjustment to a high-pressure reference state to get reliable predictions up to 1 GPa range of pressures^9^.

On the other hand, the practice of thermodynamic research and industrial applications conventionally operates with the rather simple Tait equation^10–12^, which however requires two data-fitted parameters for each isotherm. Note also that its modification^13^ leads also to the Murnaghan equation initially derived for highly compressive isotropic solids. Recently, there were some attempts to address Tait’s parameters from general thermodynamic points of view considering the ultra-high pressure range^14^ and its interpolating to the low-pressure limiting state^15^ as well as from the theory thermodynamic fluctuations^16^ that allows building the Tait-like shaped Fluctuations Theory-based Equation of State (FT-EOS), which extrapolates data obtained at ambient pressure to the elevated one up to some hundred Megapascals for a wide range of liquid’s classes, including polar, and ionic liquids and liquid mixtures^17–19^. However, the latter fails when the pressure tends to GPa range; this has been argued as the transition of liquid’s bulk modulus to the behaviour typical for isotropic solids (Murnaghan’s equation)^20^.

Note that the bulk modulus is recently considered one of the most promising thermodynamic characteristics of low-compressible media since its relative change can be treated as a small parameter^21,22^. In addition, this quantity directly relates to the behaviour of the liquid’s structure factor^23^, entropy and isochoric heat capacity^24^, and the Grüneisen parameter^25^ treated at extreme pressures by analogy to the solid state physics. Simultaneously, this analogy at saturation conditions and in the supercritical region led to the development of the phonon theory of liquid thermodynamics^26–28^.

Thus, the main problem stated in the present work is building a predictive procedure valid up to the Gigapascal range, which uses the Tait equation (in concert with the Murnaghan equation considered as an approximation of Tait’s equation at a certain range of pressures) with parameters, which require for their defining only the data measured at ambient pressure. The ingredients, which assure such a possibility is sequential considering the pressure dependence of the density and the reduced bulk modulus at varying pressure within the liner response theory combined with the ideas of the response of the medium’s oscillatory modes to the volume change.

## Results

### The procedure developed

It is demonstrated that the density of molecular liquids known for the extremely high range of elevated pressures, up to the Gigapascal range, can be predicted by the combination of two classic functional forms conventionally used only for the regression of high-pressure data. The first equation is the Tait equation

1 $$\begin{aligned} \rho ^{-1}=\rho _0^{-1}\left( 1-\frac{1}{k'}\ln \left[ 1+k'\kappa _T^0(P-P_0)\right] \right) . \end{aligned}$$

The second one is Murnagnan’s equation

2 $$\begin{aligned} \rho =\rho _0\left[ 1+k'\kappa _T^0(P-P_0)\right] ^{1/k'}. \end{aligned}$$

The predictive combination is the simple averaging

3 $$\begin{aligned} \rho _{pred}=\frac{\rho _{pred}^{Tait}+\rho _{pred}^{Murnaghan}}{2}, \end{aligned}$$

where $\rho _{pred}^{Tait}$ and $\rho _{pred}^{Murnaghan}$ are values of the density given by Eqs. (1) and (2), respectively, in which only the parameters determined at normal ambient pressure $P_0$ are used: the density $\rho _0=\rho (P_0)$, the isothermal compressibility

4 $$\begin{aligned} \kappa _T^0=\frac{1}{\rho _0c_0^2}+\frac{T\left( \alpha _P^0\right) ^2}{\rho _0C^0_P}, \end{aligned}$$

where $C_P^0$ is the isobaric heat capacity and $\alpha _P^0=-\rho _0^{-1}\left( \partial \rho _0/\partial T\right) _{P=P_0}$ is the isobaric coefficient of the thermal expansion, $c_0$ is the speed of sound at the same pressure $P_0$.

Simultaneously, Tait’ and Murnagnan’s equations themselves could serve as the estimators for the upper and lower bounds denoting possible uncertainty of the density predictions at elevated presures.

Finally, the key ingredient, which provides the predictive capacity of Eq. (3) originated from an interpretation of this parameter from the point of view of the phonon theory of liquids, is the coefficient $k'$, which has the meaning of the nonlinearity parameter determining the response of the reduced isothermal bulk modulus to the isothermal compression

5 $$\begin{aligned} k'=\left( \frac{\partial [\rho (P)\kappa _T(P)]^{-1}}{\partial P}\right) _{P=P_0}. \end{aligned}$$

It can be found as the tangent (slope) coefficient in the linear fit of the combination of the speed of sound and the density at ambient pressure $P_0$ in logarithmic coordinates

6 $$\begin{aligned} \ln \left( c_0(T)^3\rho _0(T)\right) =\textrm{const}+k\ln \left( \rho _0(T)\right) \end{aligned}$$

with two possible realisations. The first one is direct $k'=k$ which gives fractional numbers. The second one applies the rounding as follows: if *k* does not differ from the nearest integer more than 0.1 (i.e. comparable with an estimated expectation of uncertainty of well-processed reference data at the ambient pressure) then $k'=\textrm{round}(k)$, i.e. rounding to the nearest integer; otherwise, $k'=\textrm{ceil}(2k)/2$, i.e. rounding to the nearest integer of half-integer toward positive infinity.

Physically, Eq. (6) has a clear meaning within the frame of the phonon theory of liquids in the isotropic quasi-harmonic approximation. Namely, considering the high-temperature limit of Debye’s isotropic model, the characteristic frequency of the highest phonon mode at high temperatures (the range, which is considered in our work) is given by $\nu _{max}\sim c/L$, where *L* is the characteristic intermolecular distance, which can be estimated via the reciprocal cubic root of the density. Thus, Eq. (6) can be rewritten as

7 $$\begin{aligned} k'=\left( \frac{\partial \left( c_0(T)^3\rho _0(T)\right) }{\partial \left( \rho _0(T)\right) } \right) _{P=P_0} \equiv 3\left( \frac{\partial \ln \nu _{max}}{\partial \ln \rho _0} \right) _{P=P_0}=3\Gamma , \end{aligned}$$

where $\Gamma$ is the microscopic (not thermodynamic) Grüneisen’s parameter. Within this context, the rounding procedure mentioned above has an interpretation in terms of the so-called Rao’s rule, which is known as an effective regularity having a certain background in the character of oscillatory molecular motions (see the section “Discussion” for details). At the same time, this interpretation defines the limit of applicability of the proposed model: it is valid when one can neglect by the strong effects of anharmonicity and molecular diffusion in liquid state. In general, the upper bound is determined by the normal boiling temperature but in practice it is advisable to check the accuracy of the liner fit given by Eq. (6). The illustrating examples for the latter are given below and in Supplementary Material.

### Test results

**Figure 1:**
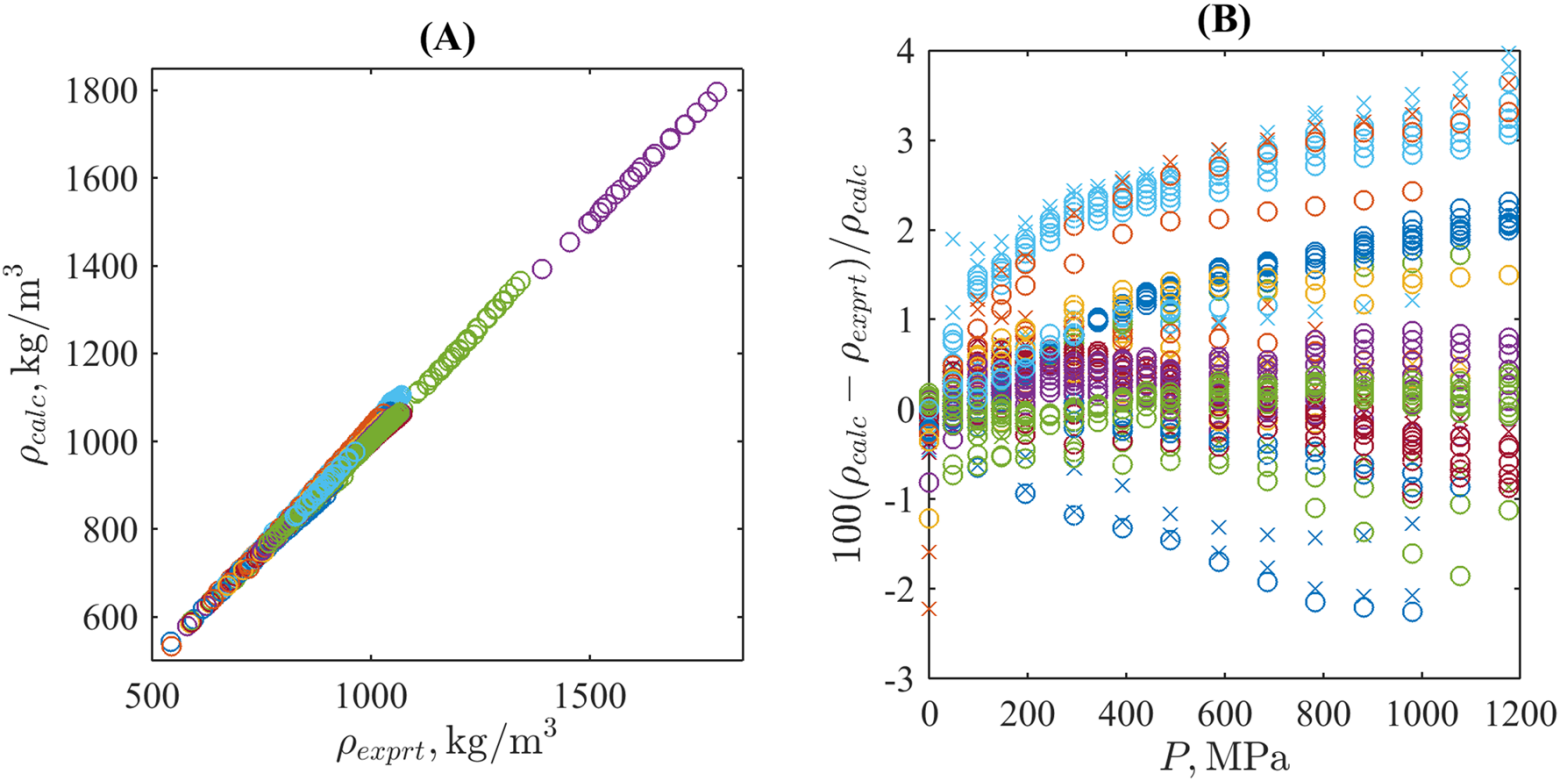
Experimental vs. predicted densities of liquids listed in Table 1 for the case of rounded isothermal nonlinearity parameters (**A**) and their relative deviations as a function of the pressure (**B**). Different colours correspond to different liquids; circles and crosses denote data for isotherms located below and above the normal boiling point.

To test the proposed method, we applied it to the classic set of high-pressure volumetric measurements by Bridgman^29^, which represents a variety of compounds belonging to different classes of molecular liquids. To calculate the nonlinearity parameter, the temperatures from $T=263.15~\textrm{K}$, which is below the lower temperature of Bridgman’s measurements, to the normal boiling point of each liquid was chosen. This allows not distinguish practically between the data along the saturation curve and at the ambient pressure. Table 1 reports results of deviations between the experimental and the predicted data for both variants of $k'$, the raw slope of the fitting straight line and its rounded version (this variant is indicated with the subsript “*r*”). Other parameters, the density and the isothermal compressibility at $P_0$ were taken for each Brigman’s temperature either from reference regressions of the REFPROP^30^ or by fitting experimental data presented in the the NIST ThermoData Engine (TDE)^31^ with the processing via Eq. (4). Since some of isotherms correspond to temperature above the normal boiling point, $P_0$, $\rho _0$ and $\kappa _T^0$ were taken at saturation conditions. These two cases are specially denoted in Table 1 and Fig. 1.

**Table 1:** The summary table of comparisons between Bridgeman’s experimental data^29^ and the densities predicted by Eqs. (3)–(2), which indicates the average (AAD) and maximal ($\mathrm {max(RD)}$) absolute relative deviations. The parameters at ambient pressure used for the predictive calculation for liquid, which names are supplied with asterisks, the correlations for the evaluated available experimental data by the NIST ThermoData Engine (TDE)^31^ were used; otherwise, the data generated by the NIST REFPRPOP^30^ were used.

Liquid	$P_{max}, ~\textrm{MPa}$	$k'$	$k'_r$	AAD, %	$\textrm{AAD}_r$, %	$\mathrm {max(RD)},~\%$	$\mathrm {max(RD}_r),~\%$
n-Pentane, $T < T_b$	981	9.82	10	1.2	1.44	1.84	2.25
n-Pentane, $T > T_b$				0.74	1.15	1.56	2.08
Isopentane, $T < T_b$	883	9.97	10	0.19	0.19	0.32	0.38
Isopentane, $T > T_b$				0.71	0.65	2.22	2.22
n-Hexane, $T < T_b$	1079	9.68	10	0.98	0.51	1.38	0.85
n-Hexane, $T > T_b$				1.39	0.56	1.74	0.86
2-Methylpentane, $T < T_b$	1177	10	10	0.11	0.11	0.29	0.3
2-Methylpentane, $T > T_b$				0.43	0.43	0.61	0.61
3-Methylpentane, $T < T_b$	1177	9.85	10	0.51	0.72	1.53	1.86
3-Methylpentane, $T > T_b$				0.09	0.4	0.3	0.87
2,2-Dimethylbutane, $T < T_b$	981	10.28	10.5	0.8	0.66	1.27	1.02
2,2-Dimethylbutane, $T > T_b$				1.28	0.86	1.99	1.21
2,3-Dimethylbutane, $T < T_b$	1079	9.82	10	0.14	0.22	0.44	0.93
2,3-Simethylbutane, $T > T_b$				0.63	0.24	0.76	0.48
n-Heptane, $T < T_b$	1079	9.93	10	0.23	0.3	0.71	0.87
n-Octane, $T < T_b$	981	9.98	10	0.54	0.51	0.95	0.92
n-Decane, $T < T_b$	785	10.06	10	0.19	0.19	0.34	0.41
Bromobenzene*, $T < T_b$	883	9.24	9.5	0.38	0.23	0.82	0.41
Chlorobenzene*, $T < T_b$	1079	10.05	10	0.71	0.75	1.61	1.72
Methyl alcohol, $T < T_b$	1177	8.36	8.5	2.44	2.21	4.13	3.65
Methyl alcohol, $T > T_b$				2.81	2.54	4.51	3.97
Ethyl alcohol, $T < T_b$	1177	9.32	9.5	0.45	0.38	0.88	0.87
Ethyl alcohol, $T > T_b$				0.65	0.44	0.91	0.77
Propyl alcohol*, $T < T_b$	1177	9.21	9.5	1.46	1.11	3.13	2.31
Isopropyl alcohol*, $T < T_b$	1177	8.95	9	1.96	1.9	3.46	3.31
Isopropyl alcohol*, $T > T_b$				2.69	2.58	3.83	3.64
n-Butyl alcohol*, $T < T_b$	1177	9.24	9.5	1.36	1.06	2.23	1.5
Isobutyl alcohol*, $T < T_b$	1177	9.46	9.5	0.39	0.34	0.98	0.87
n-Pentyl alcohol*, $T < T_b$	1177	9.31	9.5	0.3	0.13	0.87	0.4
n-Hexyl alcohol*, $T < T_b$	687	9.67	10	0.83	0.63	1.71	1.15

From Table. 1 and Fig. 1A, one can see that the proposed model allows predicting the density up to the Gigapascal range with uncertainty comparable with its expected value for these significantly high-pressure volumetric measurements. For the full set shown in this figure, the coefficient of determination $R^2=0.996$, for individual substances it varies from 0.996 to more than 0.999 (this highest $R^2$ is found for 11 and 16 from 20 total liquids in the cases of fractional and rounded $k'$, respectively). Moreover, for the majority of substances studied, the deviations are located mainly within a narrow stripe $\pm 1\%$ for the whole range of pressures, see Fig. 1B. To the number of most valuable exceptions in Table 1 belong methyl and isopropyl alcohols and, to the less extent, n-pentane and n-butyl alcohol (those sequences of markers, which tend to 4% deviations).

The case of methanol in Table 1 originates not from invalidity of the method itself but from the chosen range of data fitting common for all substances in this table. Methanol has low triple ($T=175.5~\textrm{K}$) and normal boiling ($T=337.8~\textrm{K}$) points. Thus, more correct determination of $k'$ requires a lower temperature region. This is confirmed with the test results reported below (and this also leads to $k'_r=9$ closer to other alcohols). The same is true for n-pentane, which exist in the liquid state at low temperatures (see the illustrated and discussed calculations in the section “Methods”).

An additional analysis of the choice of the fitting intervals is also provided in Supplementary Materials.

As for isopropyl alcohol, its thermodynamic parameters are not standardised due to scarcity and scattering of known experimental data, and the deviation reported in Table 1 may originate from a large uncertainty experimental data themselves. As an additional confirmation, see Fig 1B, where there are large deviations even at the reference pressure $P_0$. The case of n-butanol is similar.

**Figure 2:**
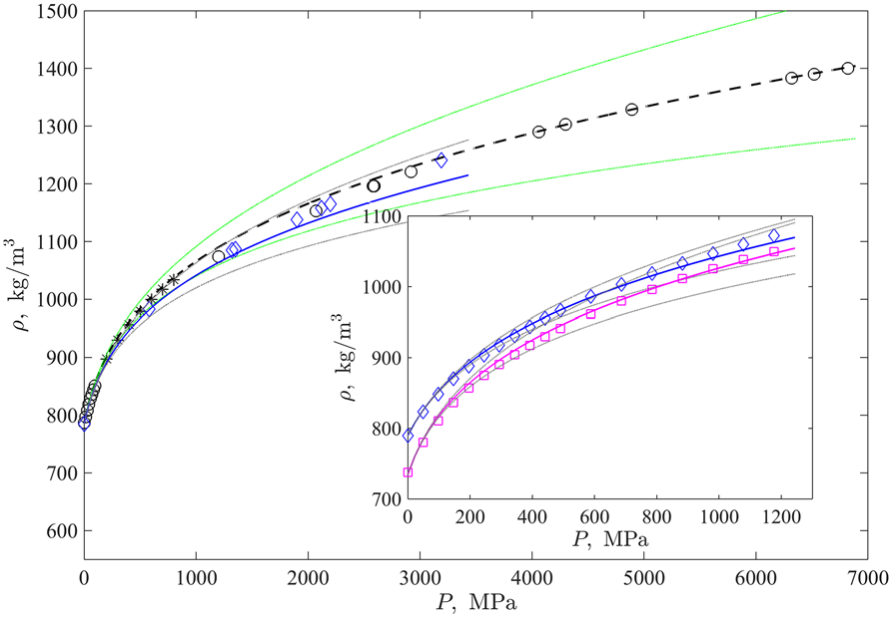
Experimental^32^ densities at $T=298.15~\textrm{K}$ for liquid methanol up to $6.82~\textrm{GPa}$ (black circles) and ethanol up to $3.19~\textrm{GPa}$ (blue diamonds) in comparison to the predicted density isotherms (black dashed and blue solid curves, respectively). To fill the gap in the region of maximal curvature of the isotherm, the data generated by the NIST REFPROP Database^30^ from $200~\textrm{MPa}$ to $800~\textrm{MPa}$ with the step $100~\textrm{MPa}$ are shown as black asterisks. Inset: Bridgman’s data^29^ for ethanol’s isotherms $T=293.15~\textrm{K}$ (blue diamonds) and $T=353.15~\textrm{K}$ (magenta squares) and the predicted isotherms shown as solid curves of the corresponding colours. Thin green (for methanol) and gray (for ethanol) lines highlight over- and under-estimation of data by the Tait and Murnaghan equations, respectively.

Apart from Bridgman’s, the data on the density of liquids under extremely high pressures (especially in the thermodynamic equilibrium state, i.e. not under shock wave compression) are extremely scarce. It is worth noting the data obtained in a diamond anvil cell^32^ for two simple alcohols, methanol and ethanol, which are important prototypic examples of liquids with significant hydrogen bonding. They are shown in comparison to the isotherms obtained by the proposed method in Fig. 2. Since, as it is discussed above, methanol has a low freezing temperature, therefore its region of solid-like molecular oscillations is shifted to lower temperatures comparing with the region used for all substances in Table 1. Thus, here we applied the linear regression according to Eq. (6) from the melting to the boiling points with the subsequent rounding of the slope that resulted in the value $k'=9.5$ coinciding now with the value for other alcohols.

One can see that the respective curves quite accurately reproduce the raw experimental data shown as markers in Fig. 2. Overall AADs for the whole isotherm are 0.47% and 1.15% for methanol and ethanol, respectively. They are within the estimated standard uncertainty of the experimental data. The maximal relative deviations are equal to 1.5% and 3.1%. Note that deviations of this range are visible for pressures of several Gigapascals for methanol, which was liquid in this experiment but this liquids state is metastable for $P>3.58~\textrm{GPa}$; for ethanol, the maximal relative deviation is detected in the point adjacent to the high-pressure freezing ($P=3.19~\textrm{GPa}$. For lower pressures, as seen in the inset in Fig. 2, the model perfectly reproduces Bridgman’s data (AADs are 0.38% and 0.43% for isotherms $T=293.15~\textrm{K}$ and $T=353.15~\textrm{K}$) which are well-coordinated with the data from^32^ within their overlapping pressure range. As one additional demonstration, one can see that the predicted curve for methanol goes through markers (asterisks) denoting the methanol’s density values reported by the NIST REFPROP^30^ for the range (200–800) $\textrm{MPA}$, $\mathrm {AAD=0.41\%}$ in this case. Respectively, Bridgeman’s isotherms for $T=293.15~\textrm{K}$, $T=303.15~\textrm{K}$ , and $T=353.15~\textrm{K}$ are with AADs equal to 0.38%, 0.47%, and 1.2% thereby correcting the values given in Table 1.

Another variant of obtaining densities of liquids at extremely high pressures is an application of shock wave compression. This process is not slow thermodynamically equilibrated and allows reaching the pressure range of up to several dozen Gigapascals without solidification. At the same time, the shock wave compression under such extremaly high pressures breaks the isothermality of the process (for example, see Ref.^33^, the wave heats n-hexane up to 5100 K at 20 GPa).

At the same time, the proposed model operates with the isothermal equation of state obtained from a general Taylor series-based expression for the pressure and the density, it should not depend on the speed of compression at moderate pressures. Moreover, the adiabatic bulk modulus characterising the sound wave and the isothermal bulk modulus are related to each other as follows from Eq. (4):

$$\begin{aligned} B_S=B_T+\frac{\alpha _P^2T}{\rho C_P}. \end{aligned}$$

At high pressures, the change of the density and the isobaric heat capacity is not drastically huge but the isothermal compressibility diminishes extremely fast as a function of the elevated pressure^34,35^ that is also captured by the FT-EoS (see Methods below) at relatively low pressures^36^, where this equation approximates both Tait’s and Murnaghan’s. Whence, at pressures higher than 1 GPa (it corresponds to the high-pressure freezing at $298.15~\textrm{K}$^37^ where the active shock-wave heating occurs, the compression route gradually changes from the isothermal to adiabatic path but the predictive parameters are expected to remain the same.

**Figure 3:**
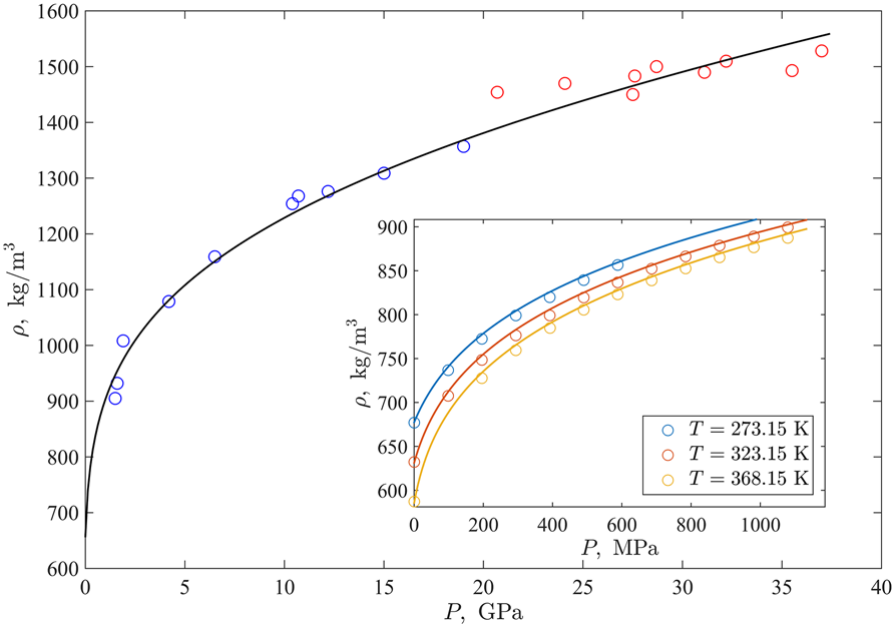
An examples of a density isotherms $T=298.15~\textrm{K}$ for n-hexane obtained via the shock wave compression^33^. Circles marking experimental values are coloured as blue for $P<20~\textrm{GPa}$ and red for $P>20~\textrm{GPa}$; between this pressures, an existence of the density jump related to a structure transition was argued in^33^. The solid line represents the model prediction based on the the half-sum of Tait’s and Murnagnan’s equations. Inset: Bridgman’s data^29^ for n-hexan’s isotherms $T=273.15~\textrm{K}$, $T=323.15~\textrm{K}$, and $T=368.15~\textrm{K}$ (circles) and the predicted isotherms shown as solid curves of the corresponding colours.

To test this, we used the experimental data on the density of shock wave-compressed up to $37~\textrm{GPa}$ n-hexane^33^ shown in Fig. 3. Note that the author argued the existence of some kind of liquid-liquid transition at about $20~\textrm{GPa}$ as the explanation for a jump in the data. For this reason, markers in the second part of the density range are coloured differently. One can see from Fig. 3 that the curve calculated with Eq. (3) up to this transition using the saturated data only for determining parameters follows the course of markers with high accuracy, which is comparable with the scattering of experimental values around a smooth curve ($AAD=1.6\%$). The inset supplies the picture with the illustration of reproducing the density at lower pressures, which are below the shock wave-originated. They are Bridgman’s data characterised in Table 1. Thus, the complete coverage of the pressure range can be stated.

Certainly, the model can not reproduce the existence of a liquid–liquid transition and the density’s jump. This explains the deviation of the course of the model predicted curve and the data points for $P>~\textrm{GPa}$. However, $AAD=1.9\%$ even in this case of (20–37) $\textrm{GPa}$ argues in favour of the utility of the proposed model for the density prediction even for such extremal high pressures.

## Discussion

The phonon theory of liquid thermodynamics^26–28^ is a modern approach quantitatively addressing the idea put forward by Frenkel^38^ that sufficiently dense liquids have certain properties, which make them closer rather to solids than to gases. First of all, it is about short-time (time intervals shorter than some characteristic relaxation time) instant oscillations of a liquid’s molecules, which resemble a disordered solid while the molecular motion during times longer than the characteristic relaxation time exhibits molecular rearrangement via diffusion-like jumps. This picture allowed the authors of the cited work to explain a number of effects related to the isochoric heat capacity of simple liquids, in particular, the transition from its value practically resembling the Dulong-Petit solids’ law at the temperatures close to the melting down to 1.5 times less value toward to high temperatures.

On the contrary, in this work, we are focused not on the energy/relaxation specificity but on the “geometric” features leading to the pressure dependence of the isothermal compressibility. Certainly, the compressibility (or the bulk modulus) of liquids and solids behaves differently as measures of the linear response to the pressure’s elevation, especially in the vicinity of the liquid-vapour coexistence line (or the ambient pressure). However, the nonlinear response of the liquid’s density changes specified by the isothermal nonlinearity coefficient (5) is found acting as the nonlinearity of compressed isotropic solids determined by their spectral characteristics defined by the microscopic Grüneisen parameter, Eqs. (6)–(7).

This parameter introduced^39^ as a measure of the response of the crystal’s lattice oscillation modes with frequencies $\nu _j$ to the change of crystal’s volume *V*

$$\begin{aligned} \Gamma _j=-\frac{\partial \ln \nu _j}{\partial \ln V} \end{aligned}$$

reduces to a single value (the right-hand side of Eq. (7) at the limit of high temperatures as observed for the high-temperature limit (leading to the Dulong–Petit law) of Debye’s theory of heat conductivity, and can be related to the compressibility (not specified respectively to the iso-property) that was first mentioned at the semi-quantitative level by Debye himself^40^ and explored in more details by Slater^41^ who discussed the response of $\nu _{max}$, as defined by the isothermal compressibility, to the volume’s change within the frames of Debye’s theory A review of these issues can be found in the work^42^.

Translating this approach to liquids, the expression connecting the change of a single vibrational frequency on the characteristic intermolecular separation was considered by Moelwyn–Hughes for the simplified model of point-wise particles oscillating in the Mie potential^43^ that resulted also in the connection of the potential’s parameters and parameters of the Murnaghan equation^44^.

In the present work, we show that specifying a particular model of intermolecular interactions is not required, and the respective isothermal nonlinearity coefficient can be practically obtained by processing the macroscopic thermodynamic parameters, the speed of sound and the density, Eq. (5).

It is worth noting that Eq. (6) can be related to another well-known empiric dependence, the so-called Rao’s rule, which reads at the ambient pressure as^45,46^

8 $$\begin{aligned} \frac{c_0^{1/3}M}{\rho _0}=R_R, \end{aligned}$$

where *M* is the molar mass and $R_R$ is a substance-specific constant known as the molecular speed of sound or the Rao constant. Actually, taking 9th power of both sides of Eq. (8) and logarithm, it reduces the form

9 $$\begin{aligned} \ln \left( c_0(T)^3\rho _0(T)\right) =9\ln \left( R_R/M\right) +10\ln \left( \rho _0(T)\right) , \end{aligned}$$

the same as Eq. (6) but with the fixed coefficient $k=10$. However, as one can see from Table 1, rounding *k* to this integer gives improvements in accuracy for the majority of non-associated liquids. Simultaneously, associated liquids require a lower value of the constant that agrees with earlier phenomenological discussions of Rao’s rule validity^47–49^.

The relation of the speed of sound to the characteristic vibrations of molecules (their frequency and energy) in a liquid as a possible origin of Eq. (8) was noted even in one of early Rao’s works^45,47^ however without any connections to isothermal compressibility changes. Later, an analogy between Rao’s rule in liquids and the Debye–Slater’s change of the isothermal compressibility connected to the microscopic Grüneisen parameter was proposed by Swamy^50^. But the cited work’s author was more focused on the value of Rao’s constant and the usage of thermodynamic Gr$\ddot{\hbox {n}}$eisen parameter (pseudo-Grüneisen parameter), which is well-applicable to isotropic solids and liquid metals but fails for molecular liquids.

On the contrary, the results of our work demonstrate that accurate predictive results can be achieved if to operate with the microscopic Grüneisen parameter directly the same as for solids. Therefore, this argues in favour of the phonon theory of liquid thermodynamics applicable when a liquid has a reactively close-packed structure (below and about the normal boiling point considering the saturation curve). In addition, it should be noted that the proposed method does not require calculating Rao’s constant because Eq. (6) operates only with the slope of the fitting straight line but not with its shift’s constant.

Finally, let us shortly discuss the combining rule given by Eq. (3). In addition to practical operability, it removes a long-discussed up to date contradiction between the applicability of Tait’s and Murnaghan’s equations^12,13,51,52^.

As shown in the work^20^, the qualitative character of the isothermal bulk modulus response to the pressure changes along an isotherm significantly depends on the molecular packing; it is better reproduced by the Murnaghan equation only for states with closely packed molecules; otherwise, the linearity of the reduced bulk modulus (see Eq. (5), leading to the FT-EoS, fulfils better. The Tait equation, taken as an interpolating phenomenological formula, balances its parameters between these two cases that may lead to deviation depending on which part of an isotherm affects the fitting procedure more.

Here we show (the detailed derivation is provided below in the Section “Methods”) that all three equations can be reduced to the same form resembling the FT-EoS with the parameter $k'$ determined at the ambient pressure (and valid for low elevated pressures) from the linear response theory. But the nonlinearity coefficient’s value of Tait’s and Murnaghan’s equations, exactly derived from their forms valid at the extra high-pressure limit, differs from the low-pressure limit in such a way that one equation “undershoots” and another “overshoots” the liner response-based value. Thus, their equally weighted average is required to get an accurate value.

## Methods

### Linear response theory

Due to low compressibility of liquids, one can operate with some first terms in the expansion of the pressure function *P*(*X*), where *X* denotes either the density $\rho$ or the specific volume $v=\rho ^{-1}$ into the Taylor series represented in the form

10 $$\begin{aligned} P=P_0+\sum _n\frac{X_0^n}{n!}\left( \frac{\partial ^n P}{\partial X^n}\right) _{T,P=P_0}\left( \frac{X-X_0}{X_0}\right) ^n \end{aligned}$$

along an isotherm. Note that it is more convenient to use the pressure as a response to the relative density/volume changes because the latter varies weakly assuring the series’ truncation mentioned above. For further processing, it is more convenient to rewrite Eq. (10) in the form, which highlights explicitly the linear and high-order terms with respect to the relative compression:

11 $$\begin{aligned} P=P_0+X_0\left( \frac{\partial P}{\partial X}\right) _{T,P=P_0}\left( \frac{X-X_0}{X_0}\right) + \sum _{n>2}\frac{X_0^n}{n!}\left( \frac{\partial ^n P}{\partial X^n}\right) _{T,P=P_0}\left( \frac{X-X_0}{X_0}\right) ^n, \end{aligned}$$

where the factor in the linear term in the right-hand side is exactly the absolute value of the isothermal bulk modulus at the pressure $P=P_0$.

Correspondingly, one can also define the response function in terms of the same series as

12 $$\begin{aligned} \left( \frac{\partial P}{\partial X}\right) _{T}=\sum _n\frac{X_0^{n-1}}{(n-1)!}\left( \frac{\partial ^n P}{\partial X^n}\right) _{T,P=P_0}\left( \frac{X-X_0}{X_0}\right) ^{n-1}. \end{aligned}$$

It is the reduced bulk modulus ${\tilde{K}}_T=\left( \rho \kappa _T\right) ^{-1}=-\left( v\kappa _T\right) ^{-1}$ when $X=\rho$ or $X=v$, respectively, and can be represented as the sum of this quantity at the ambient pressure, the linear, and the high-order terms

13 $$\begin{aligned} \left( \frac{\partial P}{\partial X}\right) _{T,P=P_0}={\tilde{K}}_T^0+\left( \frac{\partial ^2 P}{\partial X^2}\right) _{T,P=P_0}\left( \frac{X-X_0}{X_0}\right) + \sum _{n>2}\frac{X_0^{n-1}}{(n-1)!}\left( \frac{\partial ^n P}{\partial X^n}\right) _{T,P=P_0}\left( \frac{X-X_0}{X_0}\right) ^{n-1}. \end{aligned}$$

For low-compressible media like liquids, their elasticity changes weakly even in the response to high pressures. Thus, we can be limited by the second term in Eq. (13) and need to rewrite it as an explicit expression of the applied pressure. For this purpose, we express the second term in Eq. (13), linear with the relative change of *X*, as the linear function of the pressure expressed from Eq. (11) also keeping the linear term only and neglecting by the contribution of high-order terms. This results in the expression

$$\begin{aligned} \left( \frac{\partial P}{\partial X}\right) _{T,P=P_0}={\tilde{K}}_T^0+ \frac{\left( \frac{\partial ^2 P}{\partial X^2}\right) _{T,P=P_0}}{X_0\left( \frac{\partial P}{\partial X}\right) _{T,P=P_0}}\left( P-P_0\right) , \end{aligned}$$

which can be rewritten in terms of the isothermal compressibility $\kappa _T^0$ at $P=P_0$ as

14 $$\begin{aligned} \left( \frac{\partial P}{\partial X}\right) _{T,P=P_0}={\tilde{K}}_T^0\pm \left( \frac{\partial ^2 P}{\partial X^2}\right) _{T,P=P_0}\kappa _T^0\left( P-P_0\right) , \end{aligned}$$

where the signs “$+$” and “−” correspond to $X=\rho$ and $X=v$, respectively.

In the latter case, Eq. (14) looks explicitly as

15 $$\begin{aligned} \left( \frac{\partial P}{\partial v}\right) _T=-\left( v_0\kappa _T^0\right) ^{-1}\left[ 1+k'\kappa _T^0(P-P_0)\right] , \end{aligned}$$

and its integrating leads to the classic Tait’s (or, more precisely, Tait–Tammann’s) equation

16 $$\begin{aligned} v=v_0\left( 1-\frac{1}{k'}\ln \left[ 1+k'\kappa _T^0(P-P_0)\right] \right) , \end{aligned}$$

where

17 $$\begin{aligned} k'=-\frac{\left( \frac{\partial ^2 P}{\partial v^2}\right) _{T,P=P_0}}{\frac{1}{v_0}\left( \frac{\partial P}{\partial v}\right) _{T,P=P_0}} \end{aligned}$$

is a dimensionless non-linearity parameter defined in terms of isothermal reduced volume’s change, i.e. Eq. (17) provides the physical meaning of this factor in Eq. (1) in contrast to its conventional discussion as a purely phenomenological parameter. Note also that is resembles combination of volumes derivatives, which emerged in Slater’s estimations of the Grúneisen parameter of solids^41^.

On the other hand, the usage $X=\rho$ in Eq. (14) leads to

18 $$\begin{aligned} \left( \frac{\partial P}{\partial \rho }\right) _T=\left( \rho _0\kappa _T^0\right) ^{-1}\left[ 1+{\tilde{k}}'\kappa _T^0(P-P_0)\right] , \end{aligned}$$

integration of which results in the Fluctuation Theory-based Equation of State (FT-EOS)

19 $$\begin{aligned} \rho =\rho _0\left( 1+\frac{1}{{\tilde{k}}'}\ln \left[ 1+{\tilde{k}}'\kappa _T^0(P-P_0)\right] \right) \end{aligned}$$

with

20 $$\begin{aligned} {\tilde{k}}'=\frac{\left( \frac{\partial ^2 P}{\partial \rho ^2}\right) _{T,P=P_0}}{\frac{1}{\rho _0}\left( \frac{\partial P}{\partial \rho }\right) _{T,P=P_0}}. \end{aligned}$$

Note that Eqs. (16) and (19) behave closely to each other for small pressures when the logarithmic term is small if to assume $k'={\tilde{k}}'$ (within the weakly non-linear responses at pressures infinitesimally close to $P_0$) and take into account that $(1-x)^{-1}\approx 1+x,~x<<1$ [compare their forms (1) and (19)]. On the other hand, they have their own drawbacks at significantly large pressures.

First of all, the Tait equation (16) leads to zero and negative volumes after reaching some high pressure. Although such pressures are over reasonable experimental possibilities, such a non-physical behaviour is the principal physical complication. Thus, one can expect that this equation with predictive parameters, derived within the linear response theory is not well-balanced respectively to the whole possible pressure range. On the other hand, the nonlinearity parameter defined by Eq. (20) is more physically relevant because the pressure is defined as a function of growing density (Eq. (10) with $X=\rho$) is monotonously growing without sign-alternating coefficients. At the same time neglecting high-order terms within the linear response theory results in two effects: (i) the systematic underestimation of the density at extra-high pressure and (ii) the constancy of the internal pressure

$$\begin{aligned} P_i=T\frac{\alpha _P}{\kappa _T}-P \end{aligned}$$

along isotherms, while in reality, it diminished from $P_i^0=P_i(0)$ down to some minimal value and then starts to grow even overcoming the value $P_i^0$ for high pressures. Averaging over the range of pressure, where these deviations around $P_i^0$ are relatively balanced in magnitude explain the exceptional predictive properties of the FT-EoS for not extra high pressure.

However, now we need to operate with extra high pressures, where the FT-EoS is not applicable. At the same time, it is known that the experimental density at extra high pressures is almost universally well-reproduced with the Murnaghan equation, which reads as

21 $$\begin{aligned} \rho =\rho _0\left[ 1+k_M\kappa _T^0(P-P_0)\right] ^{1/k_M}. \end{aligned}$$

Its origin also can be considered as originated from some kind of the linear response theory but applied not the density/volume but directly to the bulk modulus considering is linear perturbation directly with respect to the pressure

22 $$\begin{aligned} \rho \left( \frac{\partial P}{\partial \rho }\right) _T=\left( \kappa _T^0\right) ^{-1}\left[ 1+k_M\kappa _T^0(P-P_0)\right] \end{aligned}$$

characterized by the constant parameter

23 $$\begin{aligned} k_M=\left( \frac{\partial }{\partial P}\left[ \rho \left( \frac{\partial P}{\partial \rho }\right) _T\right] \right) _T. \end{aligned}$$

### Equivalence of three isothermal equations at low elevated pressures

Note however that all three discussed equations can be approximated by the same functional expressions at not very high elevated pressures. In particular, Eq. (21) can be logarithmed

$$\begin{aligned} \ln \left( \frac{\rho }{\rho _0}\right) =k_M^{-1}\ln \left[ 1+k_M\kappa _T^0(P-P_0)\right] \end{aligned}$$

and rewritten as

24 $$\begin{aligned} \ln \left( 1+\frac{\rho -\rho _0}{\rho _0}\right) =k_M^{-1}\ln \left[ 1+k_M\kappa _T^0(P-P_0)\right] . \end{aligned}$$

For $(\rho -\rho _0)/\rho _0<<1$, since $\ln (1+x)\approx x$ for small *x*,

$$\begin{aligned} \frac{\rho -\rho _0}{\rho _0}\approx k_M^{-1}\ln \left[ 1+k_M\kappa _T^0(P-P_0)\right] \end{aligned}$$

that gives

25 $$\begin{aligned} \rho =\rho _0+ \frac{\rho _0}{k_M}\ln \left[ 1+(k_M/\rho _0)\rho _0\kappa _T^0(P-P_0)\right] , \end{aligned}$$

i.e. the form of the FT-EoS (19) with ${\tilde{k}}'=k_M$.

Simultaneously, Eq. (21) written also in terms of specific volumes as

$$\begin{aligned} \frac{v}{v_0}\equiv \left( 1+\frac{v-v_0}{v_0}\right) =\left[ 1+k_M\kappa _T^0(P-P_0)\right] ^{-1/k_M} \end{aligned}$$

and using the same approximation of logarithm for ${(v-v_0)}/{v_0}<<1$, we get, following Ref.^13^,

$$\begin{aligned} v=v_0\left( 1-k_M^{-1}\ln \left[ 1+k_M\kappa _T^0(P-P_0)\right] \right) , \end{aligned}$$

i.e. the Tait equation (16) with $k'=k_M$, which in turn, coincides with Eq. (19) within the same order of approximation.

### Isothermal nonlinearity parameter expressed from high-pressure data

However, it should be stressed that the equivalence of parameters $k'$, $k_M$, and ${\tilde{k}}'$ within the applicability of the linear response theory near the saturation curve does not fulfil in a general case.

For Murnagnan’s equation, the direct usage of the product’s derivative formula and Eq. (18) leads to

$$\begin{aligned} k_M={\tilde{k}}'+1, \end{aligned}$$

for $P=P_0$. This means that $k_M$ obtained as an empiric coefficient of the curve fitting experimental data at very high pressure should be larger than the coefficient obtained from the linear response theory at low pressure and these two values do not coincide. Thus, the substitution of the value $k'={\tilde{k}}'$ required by the linear response theory will lead to the underestimation of the density at very high pressures.

A similar conclusion can be done for the Tait equation. It is possible to express explicitly the pressure as a function of the density using the form (1),

$$\begin{aligned} P=\left( k'\kappa _T^0\right) ^{-1}\exp \left( k'\left[ 1-\frac{\rho _0}{\rho }\right] \right) -\left( k'\kappa _T^0\right) ^{-1}+P_0, \end{aligned}$$

and differentiate it twice. As a result, at the ambient pressure $P=P_0$, when $\rho =\rho _0$, the ratio of the derivatives represented by Eq. (17) gives

$$\begin{aligned} k'={\tilde{k}}'+2. \end{aligned}$$

Thus, in this case, the high-pressure-fitted parameter is also larger than the linear response-based one. But, since the Tait equation is written respectively to the specific volume, the usage of $k'={\tilde{k}}'$ instead of the empirically fitted $k'$ will result in the underestimation of the specific volume and, respectively, overestimating the density.

Thus, the effects of Murnaghan’s and Tait’s equations are opposite as highlighted in Fig. 2. However, since at low pressures both these equations should have the same course, as it is discussed above, we can simply consider their equally-weighted sum (3). Such a sum exactly reproduces the FT-EoS, which is the strict consequence of the linear response theory for the density at relatively low pressures and leads to compensating effects of high-order terms required for taking into account high compression at very high pressures, where the linear response theory is not valid.

### Correspondence between isothermal parameters calculated from the fundamental equation of state and from the saturated data

**Figure 4:**
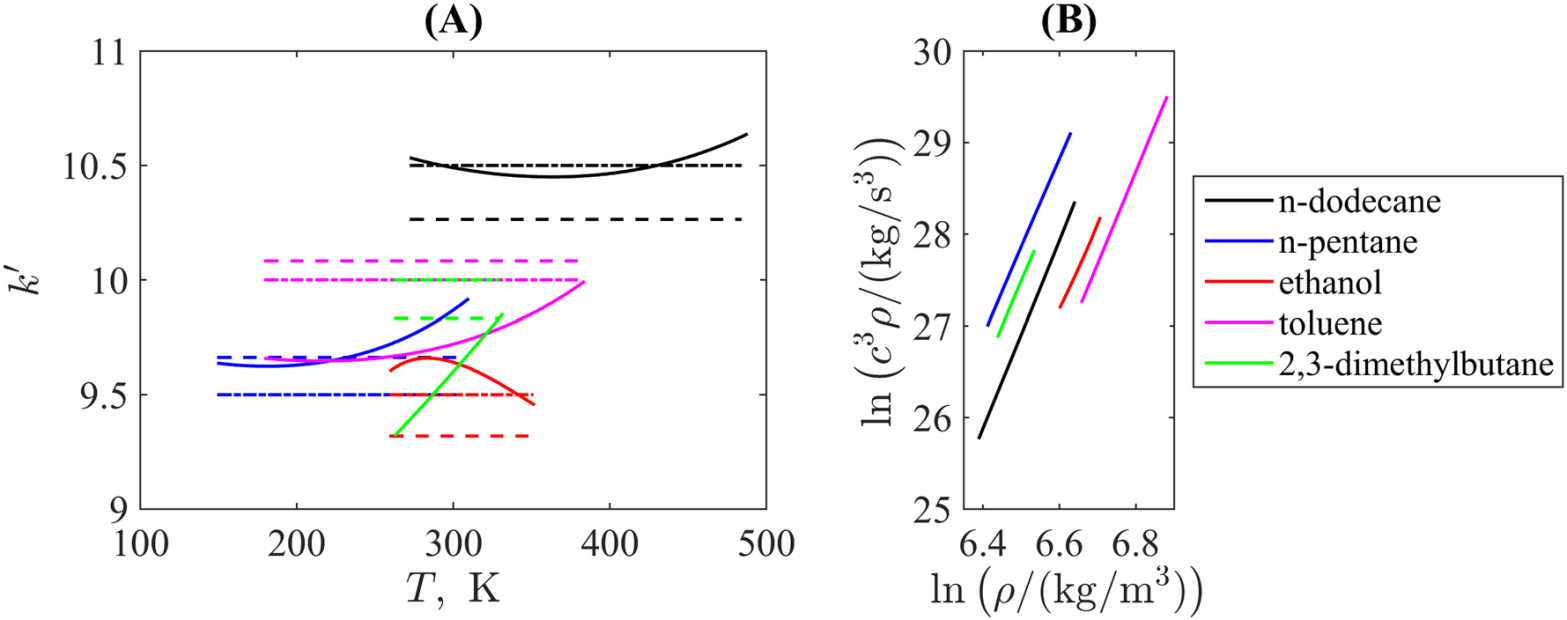
(**A**) Examples of the isothermal nonlinearity parameter from the triple point to the normal melting point for saturated molecular liquids of different classes, where solid lines are point-wise thermodynamic values calculated explicitly with derivatives of the fundamental equations of state included in the NIST REFPROP^30^, and the dashed lines are the average values defined as slopes of the linear fits of lines shown in (**B**). Liquids are marked by the same colours in both subpanels.

Figure 4A illustrates the comparison of the isothermal nonlinearity parameter $k'$ for some examples of molecular liquids belonging to different chemical classes: n-alkanes, i.e. linear hydrocarbons with different chain lengths, a highly branched isomer (2,3-dimethylbutane), typical aromatic and polar compounds (toluene and ethanol, respectively). The reference for the majority of curves is taken from the triple point to the normal boiling point along the saturation curve (that is, in principle, does not differ from values determined to the ambient pressure condition for thermodynamic parameters of the bulk liquid) are calculated directly from the definition given by Eq. (20).

The only exception is 2,3-dimethylbutane, for which the lower temperature limit is chosen at $T=263.15~\textrm{K}$ as was used for Table 1. This is connected with the properties of this branched compound, which exhibits a more complex behaviour in the region of low temperatures. The branched structure leads to specific oscillatory modes and this substance may exhibit metastability and glassy states after freezing^53^ that is also reflected in the properties of a cooled liquid. Thus, we are limited by the temperature range, which is closer to room temperatures when such anomalies are absent. Similar behaviour is noted for other branched hydrocarbons, e.g. for 3-methylpentane as additionally illustrated in Supplementary Material.

Another feature worth discussing is the isothermal nonlinearity coefficient for n-pentane, which is equal (in the rounded variant) to $$k'_r=9.5$$ in Fig. 4A in contrast to $$k'_r=10$$ in Table 1. For this lower value, $$\textrm{AAD}_r=0.75\%$$, $$\mathrm {max(RD}_r)=1.12\%$$ ($$T<T_b)$$ and $$\textrm{AAD}_r=0.43\%$$, $$\mathrm {max(RD}_r)=0.86\%$$ ($$T>T_b)$$ for Bridgman’s isotherms that is significantly better than results listed in Table 1 and confirms the statement formulated above that one needs to consider temperatures closer to the triple point for liquids with low melting/boiling temperatures to assure validity of the solid-like molecular oscillations properties required by the phonon theory.

The corresponding partial derivatives and density included in Eq. (20) for these substances are substituted as provided by the NIST REFPROP^30^ reference software from the fundamental equation of state. The values for the same source are also used to plot the dependencies (6) shown in Fig. 4B, which are sufficiently linear. The slopes of the linear fit lines of the latter are denoted in Fig. 4A as dashed straight lines and their roundings to either integer or half-integer values are dash-dotted straight lines. One can see that the latter are closer to the direct derivative-based curves, especially in the vicinity of the temperature range, for which the high-pressure density measurements are available. This supports the conclusion that such rounding having a physical background in the origin of Rao’s rule from the phonon theory of liquid thermodynamics in the quasi-harmonic approximation is plausible. In addition, such a check of linearity of the fit provides a criterion for the possibility to neglect by effects of anharmonicity and molecular diffusion, which may affect the isothermal nonlinearity parameter. Notably that the linearity in the double-logarithmic coordinates, i.e. the power-law dependence between the speed of sound and the density distinguishes liquids as more compressible substance from solids, for with such linearity fulfils between these quantities directly (Birch’s law).

It should be stressed also that the thermodynamic route of calculating $k'$ from the fundamental equation of state itself has the uncertainty about 5–7$\%$ taking into account the uncertainty of the derivative quantities (note that the same is true also for the thermodynamic route of estimating the adiabatic nonlinearity parameter, see, for example, the discussion in Ref.^54^); thus, the difference between solid curved and straight dash-dotted lines bounded within this range in Fig. 4A does not principally affect this conclusion.

### Software implementations

It should be pointed out that the proposed methods, which operate with very simple predictive equations (1)–(3) and linear regression of the reference data, Eq. (6) can be easily realised with a variety of common computational tools, even with trivial spreadsheets that makes its usage available for practical engineers.

The calculations leading to results reported in this work were done with the home-written code, which can be run with both MATLAB (any version since it uses the basic operations only) and free software GNU Octave. As of the Supporting Materials, we provide a code example (.m-file and data for 3-methylpentane) illustrating such calculations.

In addition, taking into account the growing popularity among the research community of the new free programming language Julia, we also supply in Supplementary Materials the version of code for this language as a Jupyter notebook. Its content provides an additional discussion of the procedure and its interpretation from the point of view of the temperature dependence of solid-like oscillational features affecting the isothermal nonlinearity parameter. To make this discussion available for those members of the research community who do not work with Julia–Jupyter, we also provide this notebook exported as a .pdf file containing detailed comments and illustrations.

## Conclusion

To summarise the presented results from the point of view of practical thermodynamics of liquids, let us note that utilising Bridgman’s data^29^ alongside data points and a description of the experimental procedure and possible apparatus errors, the overall uncertainty of the density determination in the experimental dataset was estimated at the level of 1%. The analysis of data from Table 1 and insets in Figs. 2, 3 shows that in the pressure range up to 1 GPa, the average (AAD) absolute relative deviations range from 0.5 to 1%. In addition, in the pressure range of up to 1 GPa, there are no liquid phase density data in the literature, apart from the data obtained by P. W. Bridgman. For lower pressures in the range up to 0.2 GPa, the uncertainty of the density obtained by direct and indirect experimental methods and their standardised systematisation for organic liquids is in the range of 0.1–0.5%^55^, which allows us to propose the usefulness of the derived equation of state model for the construction and validation of future measuring instruments for measuring density at pressures up to 1 GPa. Thus, it could be expected that the recommended model, which does not require preliminary high-pressure measurements to get its coefficients, could be reliably applied to validate the measured density of a number of other compressed classical liquids in broad ranges of temperatures and pressures up to 1 GPa.

On the other hand, the model’s construction, which addresses the characteristic frequency of intermolecular oscillation mimicking the limiting frequency of Debye’s theory of heat conductivity of solids, shed light on another possible application of the phonon theory of liquids distinct from purely energetic/heat capacity issues studied before. Moreover, this route based on the linear response theory being combined with thermodynamic equalities may open new perspectives for the predictive modelling of another thermodynamic functions in the single phase region of liquids under high pressures as well.

## Supplementary Information

Supplementary Information.

## Data Availability

Testing the proposed model was carried out with the already published data, sources of which are referenced in captions to Table 1 and figures. The computational code implementing the model is provided as Supplementary Material.
