# Supplementary material for: Combining the Tait equation with the phonon theory allows predicting the density of liquids up to the Gigapascal range
Source: Sci Rep. 2023 Mar 7;13:3766. doi: 10.1038/s41598-023-30917-0 (PMC9992665; doi:10.1038/s41598-023-30917-0)
Supplement: Supplementary file 1 — Supplementary Information. [file 41598_2023_30917_MOESM1_ESM.zip › Supplementary_pdf_Julia.pdf]

An example: the comparison of the predicted and experimental densities for 3-methylpentane

In [23]:
```
using CSV, DataFrames, Polynomials, Statistics, Plots, LaTeXStrings, Colors, ColorSchemes, Printf
```

Isothermal equations of state and their predictive combination

These three functions define Tait's and Murnaghan's equations as well as their half-sum, which is used to predict the density at elevated pressures. As input parameters, they require the initial pressure, density, isothermal compressibility and the isothermal nonlineaity parameter for a given isotherm as well as an array specifying the set of elevated pressures.

In [24]:
```
Tait(P,P0,rho0,kappa0,k) = rho0 ./ (1 - k^(-1) .* log.(k * kappa0 .* (P-P0) .+ 1));
```

In [25]:
```
Murnaghan(P,P0,rho0,kappa0,k) = rho0 .* (1+k .* kappa0 .* (P .- P0)).^(1/k);
```

In [26]:
```
rho_pred(P,P0,rho0,kappa0,k) = (Tait(P,P0,rho0,kappa0,k) .+ Murnaghan(P,P0,rho0,kappa0,k)) ./ 2;
```

Thermodynamic parameters at ambient pressure

There are different possibility to get thermodynamic quantities at the ambient (or saturated) pressure. Here the fundamental equation of state is used due to its existence for 3-methylpentane. The file repopting its parameters in .fld format is free availiable as supplemental to the article [Gao, Wu & Lemmon, *J. Chem. Eng. Data* **50** (2021) 033103 (https://aip.scitation.org/doi/suppl/10.1063/1.5093644)]. This file should be placed to the folder FLUIDS of the installed free version of REFPROP, mini-REFPROP - Version 10.0 (https://trc.nist.gov/refprop/MINIREF/MINIREF.HTM) that makes availiable to compute and save to a text file all required thermodynamic quantities. In that file the saturated data from the melting point to the temperature slightly higher than the normal boiling point ($T = 336.15\ \mathrm{K}$) are used (the dimensions are stated in the text file).

In [27]:
```
Tbl0 = CSV.read("3methylpentane_sat_REFPROP.txt", DataFrame; delim = '\t', skipto = 5, header = 3)
```

Out[27]: 254 rows × 8 columns (omitted printing of 1 columns)

	Temperature	Pressure	Density	Density_1	Sound Speed	Sound Speed_1	Isothrm. Compress.
	Float64	Float64	Float64	Float64	Float64	Float64	Float64
1	120.15	6.71e-12	821.68	5.7883e-10	1907.3	113.92	0.00046158
2	121.15	9.391e-12	820.68	8.0341e-10	1902.1	114.36	0.00046458
3	122.15	1.3064e-11	819.69	1.1085e-9	1897.0	114.8	0.00046761
4	123.15	1.8066e-11	818.7	1.5205e-9	1891.9	115.24	0.00047065
5	124.15	2.4841e-11	817.71	2.0738e-9	1886.8	115.68	0.00047371
6	125.15	3.3964e-11	816.72	2.8128e-9	1881.7	116.12	0.0004768
7	126.15	4.6186e-11	815.74	3.7946e-9	1876.6	116.55	0.0004799
8	127.15	6.247e-11	814.76	5.0922e-9	1871.5	116.98	0.00048302
9	128.15	8.4058e-11	813.78	6.7985e-9	1866.4	117.41	0.00048617
10	129.15	1.1253e-10	812.8	9.031e-9	1861.3	117.84	0.00048933
11	130.15	1.4991e-10	811.83	1.1938e-8	1856.3	118.27	0.00049252
12	131.15	1.9873e-10	810.85	1.5706e-8	1851.2	118.7	0.00049572
13	132.15	2.6222e-10	809.88	2.0566e-8	1846.2	119.12	0.00049895
14	133.15	3.4439e-10	808.91	2.6808e-8	1841.1	119.54	0.0005022
15	134.15	4.5028e-10	807.94	3.4789e-8	1836.1	119.96	0.00050547
16	135.15	5.8615e-10	806.98	4.4951e-8	1831.1	120.38	0.00050876
17	136.15	7.5973e-10	806.01	5.7835e-8	1826.1	120.8	0.00051208
18	137.15	9.8059e-10	805.05	7.4104e-8	1821.1	121.22	0.00051541
19	138.15	1.2605e-9	804.09	9.4565e-8	1816.1	121.63	0.00051877
20	139.15	1.6137e-9	803.13	1.202e-7	1811.1	122.05	0.00052216
21	140.15	2.0579e-9	802.17	1.5219e-7	1806.1	122.46	0.00052556
22	141.15	2.6143e-9	801.22	1.9197e-7	1801.2	122.87	0.00052899
23	142.15	3.3087e-9	800.27	2.4125e-7	1796.2	123.28	0.00053245
24	143.15	4.1722e-9	799.31	3.0208e-7	1791.2	123.69	0.00053593
25	144.15	5.2422e-9	798.36	3.7692e-7	1786.3	124.09	0.00053943
26	145.15	6.5634e-9	797.42	4.6867e-7	1781.4	124.5	0.00054295
27	146.15	8.1895e-9	796.47	5.8078e-7	1776.4	124.9	0.00054651
28	147.15	1.0184e-8	795.52	7.1732e-7	1771.5	125.31	0.00055008
29	148.15	1.2623e-8	794.58	8.831e-7	1766.6	125.71	0.00055369
30	149.15	1.5596e-8	793.64	1.0837e-6	1761.7	126.11	0.00055732
⋮	⋮	⋮	⋮	⋮	⋮	⋮	⋮

Data for the liquid state are extracted from the full dataframe.

In [28]:
```
T0=Tbl0[:,1];# K
P0=Tbl0[:,2];# MPa
rho0=Tbl0[:,3];# kg/m^3
c0=Tbl0[:,5];# m/s
kappaT0=Tbl0[:,7];# 1/MPa
```

Experimental data at elevated presures

The experimental data for 3-methylpentane compressed up to more than 1 GPa were taken from the free accessible Bridgman's article [*Proc. Am. Acad. Arts Sci.* **66** (1931) 185–233 (https://www.jstor.org/stable/20026332)]. It reports relative isothermal changes of the volume along isotherms reduced to the specific volume at $T = 273.15\ \mathrm{K}$; thus, the density was calculated using the REFPROP-based reference density value.

In [29]:
```
Tblexp = CSV.read("Bridgman_3methylpentane.txt", DataFrame; delim = '\t', skipto = 4, header = 2)
```
Out[29]: 42 rows × 3 columns

	T	P	rho
	Float64	Float64	Float64
1	273.15	0.101	682.22
2	273.15	49.0	723.1
3	273.15	98.1	748.0
4	273.15	147.0	768.3
5	273.15	196.0	785.1
6	273.15	294.0	811.3
7	273.15	392.0	831.9
8	273.15	490.0	850.4
9	273.15	588.0	867.3
10	273.15	686.0	883.3
11	273.15	784.0	898.6
12	273.15	882.0	912.5
13	273.15	980.0	925.4
14	273.15	1078.0	937.6
15	323.15	0.101	635.9
16	323.15	49.0	690.2
17	323.15	98.0	720.3
18	323.15	147.0	742.4
19	323.15	196.0	760.4
20	323.15	294.0	790.5
21	323.15	392.0	814.2
22	323.15	490.0	833.1
23	323.15	588.0	850.0
24	323.15	686.0	864.9
25	323.15	784.0	879.0
26	323.15	882.0	892.1
27	323.15	980.0	904.3
28	323.15	1078.0	915.1
29	323.15	1176.0	925.3
30	368.15	98.1	695.1
⋮	⋮	⋮	⋮

Determining temperatures of the isotherms contained in the experimental data

In [30]:
```
T = unique(Tblexp[:,1]);
```

Calculations illustrated

The average nonlineaity parameter

The value of this key parameter was determined by the linear regression within the range of temperatures slightly extending the the interval of the experimentally studied isotherms. Thus, the linear regression, which gives the nonlinearity parameter k' denoted as k_, has the followng form

In [31]:
```
fit_int = (T0 .> (T[1] - 10)) .& (T0 .< (T[end] + 10));
```

In [32]:
```
pk=fit(log.(rho0[fit_int]),log.(c0[fit_int] .^3 .* rho0[fit_int]),1);
```

In [33]:
```
k_ = pk[1]
```

Out[33]:
```
10.073515072940674
```

In [34]:
```
plot(log.(rho0[fit_int]),log.(c0[fit_int] .^3 .* rho0[fit_int]);
      marker = :circle, seriestype=:scatter, color = :red, markerstrokecolor = :darkred,
      label = "REFPROP data (fitted)")
plot!(log.(rho0[.~fit_int]),log.(c0[.~fit_int] .^3 .* rho0[.~fit_int]);
      marker = :circle, seriestype=:scatter, color = :white, markerstrokecolor = :darkred,
      label = "REFPROP data (not fitted)")
plot!(log.(rho0),pk.(log.(rho0)); color = :blue, linewidth = 2, label = "Linear fit")
plot!(xlabel = L"\ln(\rho/\mathrm{kg/m^3})", ylabel = L"\ln\left(c^3\rho/(\mathrm{kg/s^3})\righ
t)",
      fontfamily="Computer Modern", guidefontsize = 14, xtickfontsize=12, ytickfontsize=12,
      legend=:bottomright)
```

Out[34]:

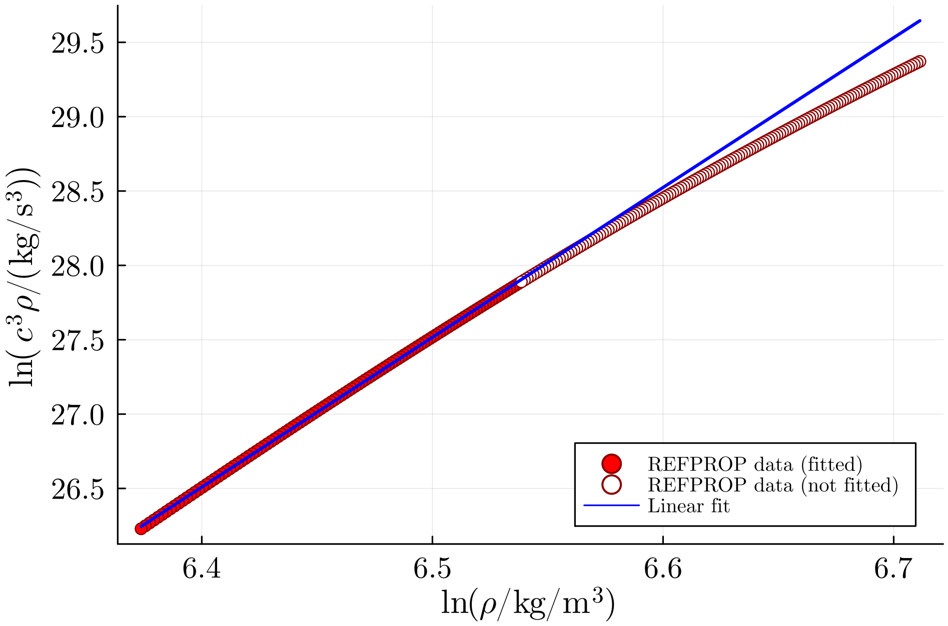

It shlould be pointed out that 3-methylpentane has an extremely low freezing temperature (from $T = 110$ K to $T = 155$ K accordingly to differrent experimental studies; REFPROP defines the liquid-vapour coexistence curves as starting from $T = 120$ K) and experimental data for the low temparature interval are very scarse. Therefore, the values reported by the REFPROP for very low temperatures are mainly extrapolations. Thus, the deviation from the linear regression there should not be considered as as deserving of special discussion.

At the same time, the linear regression within the fitted interval of temparatures lloks reasonably. It should be pointe out that the third Bridgman's isotherm ($T = 368.15$ K) is above the normal boiling point ($T = 336.15$ K), and the liquid's structure is more sparse there that leads to the less relevant its consideration from the point of view of the solid-like phonon thermodynamics.

Predicting the density

Below, after the calculation/plotting loop, the avearage absolute relative deviations (AAD) between the experimental and the predicted data are shown for all isotherms as well as the plots, where circles denote experimental data and curves are model calculations.

Note also that as discussed in the main text of the paper, better results, which also have certain background in the character of molecular oscillations, can be achived by rounding the slope of the fitting straight line to the nearest integer (or half-integer) number as follows:

In [35]:
```
if abs(k_-round(k_))<=0.1
    k_=round(k_);
else
    k_=ceil(2*k_)/2;
end
```

Out[35]: 10.0

In [36]:
```
P = plot()
RD = zeros(length(T),1); # Initializing an array of relative deviations
import ColorSchemes.tol_light
for j in 1:length(T)
    rho_exp = Tblexp.rho[Tblexp.T .== T[j]];
    plot!(P, Tblexp.P[Tblexp.T .== T[j]], rho_exp;
            marker = :circle, seriestype=:scatter, label = string(T[j])*" K", color=tol_light[j])
    rho_calc = rho_pred.(Tblexp.P[Tblexp.T .== T[j]],
                       P0[floor.(T0) .== floor(T[j])],
                       rho0[floor.(T0) .== floor(T[j])],
                       kappaT0[floor.(T0) .== floor(T[j])],k_);
    display(@sprintf("AAD(%.2f", T[j]) * " K)=" * @sprintf("%.2f", mean(100 * abs.(rho_exp-rho_cal
c) ./ rho_exp)) *  "%")
    plot!(P, Tblexp.P[Tblexp.T .== T[j]], rho_calc;
        linewidth = 2, label = :none, color=tol_light[j])
end
plot!(P,xlabel = L"P,~\mathrm{MPa}", ylabel = L"\rho,~\mathrm{kg/m^3}",
      fontfamily="Computer Modern", guidefontsize = 14, xtickfontsize=12, ytickfontsize=12,
      legend=:bottomright)
display(P)
```

"AAD(273.15 K)=0.77%"

"AAD(323.15 K)=0.68%"

"AAD(368.15 K)=0.43%"

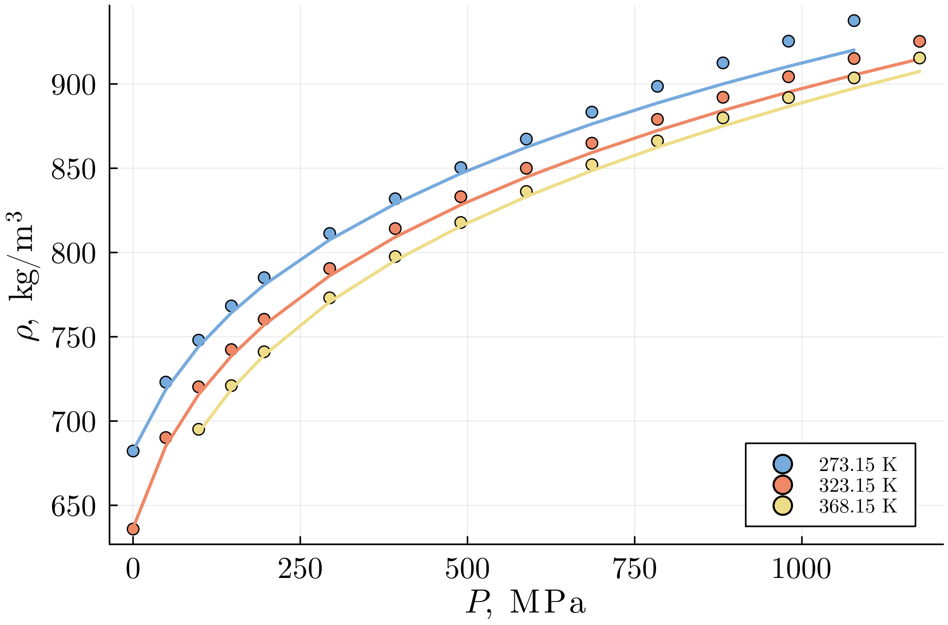

One can see that this value of the nonlinearity parameter k' obteined by avaraging fit over a wide range of temparatures gives reasonable and relatively uniformly behaving deviation for all isotherms, in principle, comparable with expeimental uncertianty for measurements at such high pressures.

The next figure illustrates how this resulting prediction curve relates to the components of the model, i.e. Tait's and Murnaghan's equations.

In [37]:
```
P = plot()
import ColorSchemes.tol_light
for j in 1:length(T)
    rho_exp = Tblexp.rho[Tblexp.T .== T[j]];
    plot!(P, Tblexp.P[Tblexp.T .== T[j]], rho_exp;
             marker = :circle, seriestype=:scatter, label = string(T[j])*" K", color=tol_light[j])
    rho_calc = rho_pred.(Tblexp.P[Tblexp.T .== T[j]],
                         P0[floor.(T0) .== floor(T[j])],
                         rho0[floor.(T0) .== floor(T[j])],
                         kappaT0[floor.(T0) .== floor(T[j])],k_);
     rho_calc_Tait = Tait.(Tblexp.P[Tblexp.T .== T[j]],
                         P0[floor.(T0) .== floor(T[j])],
                         rho0[floor.(T0) .== floor(T[j])],
                         kappaT0[floor.(T0) .== floor(T[j])],k_);
    rho_calc_Murnaghan = Murnaghan.(Tblexp.P[Tblexp.T .== T[j]],
                         P0[floor.(T0) .== floor(T[j])],
                         rho0[floor.(T0) .== floor(T[j])],
                         kappaT0[floor.(T0) .== floor(T[j])],k_);
    plot!(P, Tblexp.P[Tblexp.T .== T[j]], rho_calc;
        linewidth = 2, label = :none, color=tol_light[j])
     plot!(P, Tblexp.P[Tblexp.T .== T[j]], rho_calc_Tait;
        linestyle=:dash, linewidth = 1, label = :none, color=tol_light[j])
     plot!(P, Tblexp.P[Tblexp.T .== T[j]], rho_calc_Murnaghan;
        linestyle=:dashdot, linewidth = 1, label = :none, color=tol_light[j])
end
plot!(P,xlabel = L"P,~\mathrm{MPa}", ylabel = L"\rho,~\mathrm{kg/m^3}",
      fontfamily="Computer Modern", guidefontsize = 14, xtickfontsize=12, ytickfontsize=12,
      legend=:bottomright)
display(P)
```

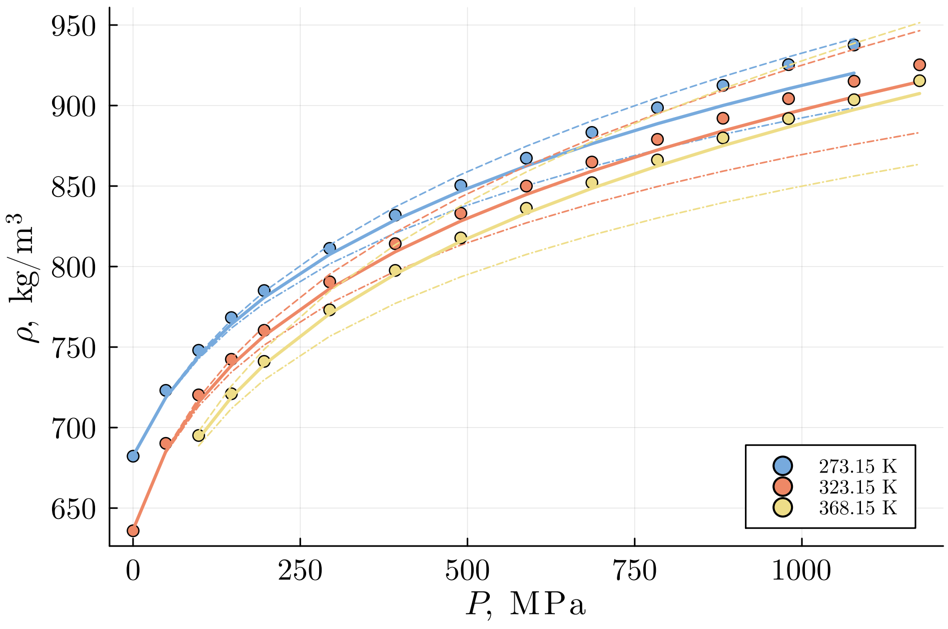

One can see that the Murnagnan equation (dash-dotted curves) underestimate the density while the Tait equation overestimates it. Their half-sum goes closer to the experimantal data.

Further, it is worth investigating the temeparature dependence of the nonlineaity parameter k' taking into account the change momolecular oscillational properties in such a wide range of temperatures. For this reason, the next illustration operates not with a global linear regression in a wide temperature range but with a *local* variant: the speeds of sound and the density are chose in the interval $\pm 20\ \mathrm{K}$ around each experimental isotherm.

In [38]:
```
P = plot()
import ColorSchemes.tol_light
for j in 1:length(T)
    rho_exp = Tblexp.rho[Tblexp.T .== T[j]];
    plot!(P, Tblexp.P[Tblexp.T .== T[j]], rho_exp;
             marker = :circle, seriestype=:scatter, label = string(T[j])*" K", color=tol_light[j])
    pkj=fit(log.(rho0[(T0 .> (T[j]-20)) .& (T0 .< (T[j]+20))]),
            log.(c0[(T0 .> (T[j]-20)) .& (T0 .< (T[j]+20))] .^3 .* rho0[(T0 .> (T[j]-20)) .& (T0 .
< (T[j]+20))]),1);
    rho_calc = rho_pred.(Tblexp.P[Tblexp.T .== T[j]],
                         P0[floor.(T0) .== floor(T[j])],
                         rho0[floor.(T0) .== floor(T[j])],
                         kappaT0[floor.(T0) .== floor(T[j])],pkj[1]);
    display(@sprintf("AAD(%.2f", T[j]) * " K)=" * @sprintf("%.2f", mean(100 * abs.(rho_exp-rho_cal
c) ./ rho_exp)) *  "%")
    plot!(P, Tblexp.P[Tblexp.T .== T[j]], rho_calc;
        linewidth = 2, label = :none, color=tol_light[j])
end
plot!(P,xlabel = L"P,~\mathrm{MPa}", ylabel = L"\rho,~\mathrm{kg/m^3}",
      fontfamily="Computer Modern", guidefontsize = 14, xtickfontsize=12, ytickfontsize=12,
      legend=:bottomright)
display(P)
```

```
"AAD(273.15 K)=0.33%"

"AAD(323.15 K)=0.84%"

"AAD(368.15 K)=1.52%"
```

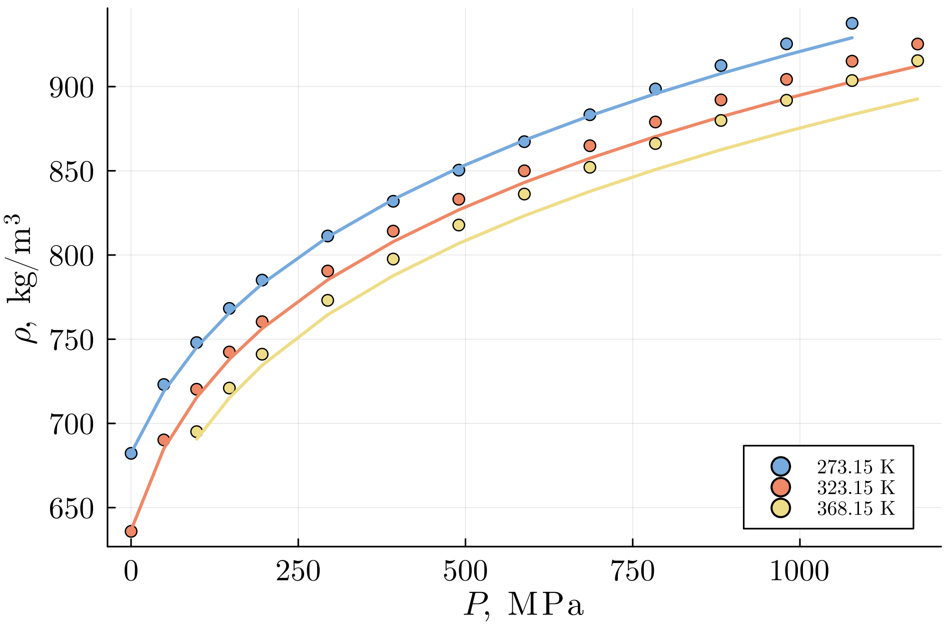

Here one can see a significant improvement of the prediction's accuracy for the "cold" isotherms $T = 273.15$ K, the result comparable with the previous one for $T = 323.15$ K (13 K below the normal boiling point) and worsening the accuracy for the "hot" isotherm $T = 368.15$ K (32 K above the normal boiling point). These behaviour argues in favour of the interpretation based on the phonon theory of liquids because only the "cold" liquids can be can be reasonably considered as similar to solid states with respect to their oscillational (phonon) properties.

Additional discussions and comparisons

Rough esxtimations: predicting the density using volumetric and acoustic data only

It can be noted that the considered combination of $c^3\rho$ and a function of ρ can be put in direct comparison to the well-known empirical Rao's rule, which reads $c^{1/3}M/\rho = R_{Rao}$, where M is the molar mass and R_{Rao} is the so-called molacular speed of sound (Rao's constant specific for a liquid). This expression can be revritten as $c^3\rho = (R_{Rao}/M)^3\rho^{10}$, from which it follows that the considered nonlinearity parameter is equal to $k'_{Rao} = 10$ that is close to the global fitting result obtained above.

In addition, it should be noted that for majority of polyatomic molecualar liquids the heat capacity ratio $C_p/C_v = \kappa_T/\kappa_S$, equal to the ratio of the isothermal and adiabatic compressibilities, is close to $1.2 - 1.3$. These values can be used as an avarage approximation even for the processing data obtained with the diamond anvil cell, see e.g. [Nakamura, Fujishiro, & Tamura, JSME Int. J. Ser. C., **38** (1995) 122-127 (https://doi.org/10.1299/jsmec1993.38.122)]. Thus, if we replace the isothermal compressiblity by the value approximated using the adiabatic one obtained from the speed of sound and the density ($\kappa_T^0 \approx 1.2/(c^2\rho)$), and subtitute Rao's rule-based $k' = 10$, we get the following picture:

In [39]:
```
P = plot()
import ColorSchemes.tol_light
for j in 1:length(T)
    rho_exp = Tblexp.rho[Tblexp.T .== T[j]];
    plot!(P, Tblexp.P[Tblexp.T .== T[j]], rho_exp;
             marker = :circle, seriestype=:scatter, label = string(T[j])*" K", color=tol_light[j])
    pkj=fit(log.(rho0[(T0 .> (T[j]-20)) .& (T0 .< (T[j]+20))]),
            log.(c0[(T0 .> (T[j]-20)) .& (T0 .< (T[j]+20))] .^3 .* rho0[(T0 .> (T[j]-20)) .& (T0 .
< (T[j]+20))]),1);
    rho_calc_1_2 = rho_pred.(Tblexp.P[Tblexp.T .== T[j]],
                       P0[floor.(T0) .== floor(T[j])],
                       rho0[floor.(T0) .== floor(T[j])],
                       1.2e6 ./ (rho0[floor.(T0) .== floor(T[j])] .* c0[floor.(T0) .== floor(T
[j])].^2),10);
     rho_calc_1_3 = Tait.(Tblexp.P[Tblexp.T .== T[j]],
                       P0[floor.(T0) .== floor(T[j])],
                       rho0[floor.(T0) .== floor(T[j])],
                       1.3e6 ./ (rho0[floor.(T0) .== floor(T[j])] .* c0[floor.(T0) .== floor(T
[j])].^2),10);
    display(@sprintf("AAD(%.2f", T[j]) * " K)=" * @sprintf("%.2f", mean(100 * abs.(rho_exp-rho_cal
c_1_2) ./ rho_exp)) *
        "% (γ=1.2) " * @sprintf("%.2f", mean(100 * abs.(rho_exp-rho_calc_1_3) ./ rho_exp)) * "% (γ
=1.3) ")
    plot!(P, Tblexp.P[Tblexp.T .== T[j]], rho_calc_1_2;
        linewidth = 2, label = L"\gamma=1.2", color=tol_light[j])
    plot!(P, Tblexp.P[Tblexp.T .== T[j]], rho_calc_1_3;
         linestyle = :dash, linewidth = 2, label = L"\gamma=1.3", color=tol_light[j])
end
plot!(P,xlabel = L"P,~\mathrm{MPa}", ylabel = L"\rho,~\mathrm{kg/m^3}",
      fontfamily="Computer Modern", guidefontsize = 14, xtickfontsize=12, ytickfontsize=12,
      legend=:bottomright)
display(P)
```

```
"AAD(273.15 K)=1.50% (γ=1.2) 0.43% (γ=1.3) "

"AAD(323.15 K)=1.35% (γ=1.2) 1.23% (γ=1.3) "

"AAD(368.15 K)=1.18% (γ=1.2) 2.64% (γ=1.3) "
```

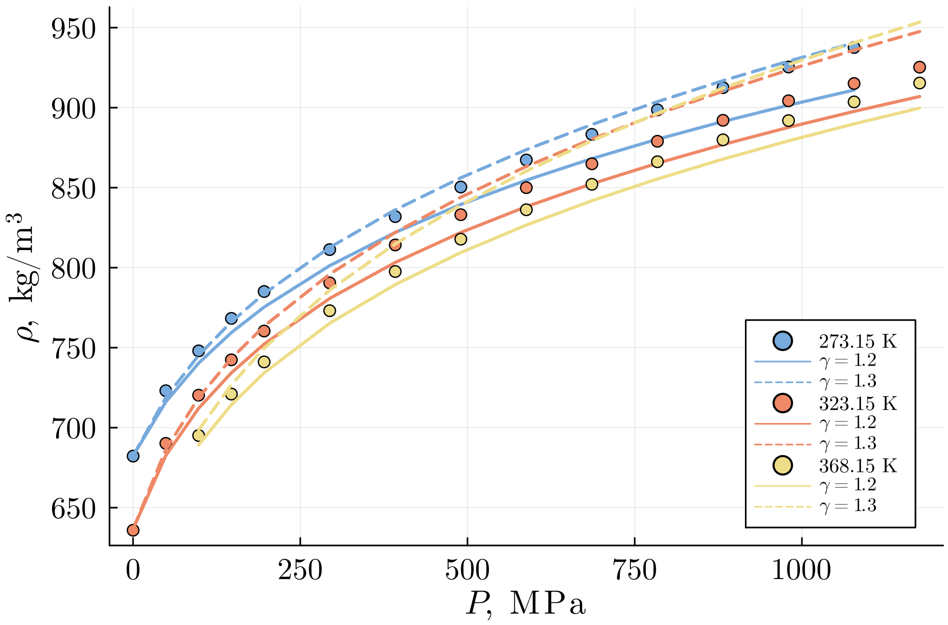

The deviations are reduced to the very acceptable values comparable with the experimental uncertainty at least for not extra high pressures and below the boiling point. Thus, this opens opportunities to apply the proposed model for operating also with substances, for which the reference data at the ambinet pressure absent or scarce, e.g. newly synthesized compounds.

```
In [ ]:
```
